# Potential impact of host immunity on malaria treatment outcome in Tanzanian children infected with *Plasmodium falciparum*

**DOI:** 10.1186/1475-2875-6-153

**Published:** 2007-11-16

**Authors:** Anders Enevold, Watoky MMM Nkya, Michael Theisen, Lasse S Vestergaard, Anja TR Jensen, Trine Staalsoe, Thor G Theander, Ib C Bygbjerg, Michael Alifrangis

**Affiliations:** 1 Centre for Medical Parasitology, Institute of International Health, Immunology and Microbiology, University of Copenhagen, Copenhagen, Denmark; 2 Mbeya Referral Hospital (MRH), Mbeya, Tanzania; 3 Department of Infectious Disease Immunology, Statens Serum Institute, Copenhagen, Denmark; 4 Department of Epidemiology, Statens Serum Institute, Copenhagen, Denmark

## Abstract

**Background:**

In malaria endemic areas children may recover from malaria after chemotherapy in spite of harbouring genotypically drug-resistant *Plasmodium falciparum*. This phenomenon suggests that there is a synergy between drug treatment and acquired immunity. This hypothesis was examined in an area of moderately intense transmission of *P. falciparum *in Tanzania during a drug trail with sulphadoxine-pyrimethamine (SP) or amodiaquine (AQ).

**Methods:**

One hundred children with uncomplicated malaria were treated with either SP or AQ and followed for 28 days. Mutations in parasite genes related to SP and AQ-resistance as well as human sickle cell trait and alpha-thalassaemia were determined using PCR and sequence-specific oligonucleotide probes and enzyme-linked immunosorbent assay (SSOP-ELISA), and IgG antibody responses to a panel of *P. falciparum *antigens were assessed and related to treatment outcome.

**Results:**

Parasitological or clinical treatment failure (TF) was observed in 68% and 38% of children receiving SP or AQ, respectively. In those with adequate clinical and parasitological response (ACPR) compared to children with TF, and for both treatment regimens, prevalence and levels of anti-Glutamate-rich Protein (GLURP)-specific IgG antibodies were significantly higher (P < 0.001), while prevalence of parasite haplotypes associated with SP and AQ resistance was lower (P = 0.02 and P = 0.07, respectively). Interestingly, anti-GLURP-IgG antibodies were more strongly associated with treatment outcome than parasite resistant haplotypes, while the IgG responses to none of the other 11 malaria antigens were not significantly associated with ACPR.

**Conclusion:**

These findings suggest that GLURP-specific IgG antibodies in this setting contribute to clearance of drug-resistant infections and support the hypothesis that acquired immunity enhances the clinical efficacy of drug therapy. The results should be confirmed in larger scale with greater sample size and with variation in transmission intensity.

## Background

*Plasmodium falciparum *resistance to commonly available antimalarial drugs such as chloroquine (CQ), amodiaquine (AQ) sulphadoxine-pyrimethamine (SP) is now widespread in most malaria-endemic areas, including Tanzania [1,2]. It has been established that polymorphisms in the parasite dihydrofolate reductase (*dhfr*), dihydropteroate synthetase (*dhps*) and chloroquine resistance transporter (*Pfcrt*) genes are associated with SP and CQ resistance, respectively in vitro [3,4]. Point mutations at positions N51I, C59R and S108N in the *dhfr *gene [5,6] and at positions A437G and K540E in the *dhps *gene [7,8] have shown to predict a reduced efficacy to SP in vivo. Likewise, the K76T mutation in the *Pfcrt *gene is a well described predictor of reduced parasite susceptibility to CQ [9], and to a lesser extent AQ [10]. The prevalence of these mutations has increased as a result of high drug pressure in most sub-Saharan countries in recent years (reviewed in [2,11]).

Patients infected with *P. falciparum *parasites carrying such drug-resistant mutations sometimes overcome infection after treatment [12]. The ability to recover has been associated with host age [13,14] and transmission intensity [15,16], reflecting an effect of acquired host immunity. From animal models it has also been established that immunity enhances the efficacy of malaria drug treatment [17]. Moreover, haemoglobinopathies, such as sickle cell trait, has been related to increased efficacy of SP treatment of uncomplicated falciparum malaria in Kenya [18]. Therefore, recovery from malaria may depend on the drug efficacy and parasite drug-resistance, as well as a complex interaction with host factors like acquired immunity and innate resistance e.g. haemoglobinopathies.

Various studies have investigated the relationship between potential immune mechanisms, such as antibody responses, and therapeutic efficacy. It has been demonstrated that increased amounts of anti-RESA and anti-NANP antibodies in patients treated with CQ were associated with better clearance of resistant parasites [19,20], whereas other studies could not establish evidence for elevated anti-MSP1 and anti-AMA1 antibody levels in patients recovering after treatment with CQ, SP or AQ [21-23]. These observations are however difficult to compare, when factors like patient age, innate resistance, intensity of transmission and level of drug resistance vary substantially between these studies and may influence treatment outcome.

The objective of this study was to evaluate factors influencing outcome of antimalarial treatment to uncomplicated *P. falciparum *malaria, such as acquired immunity, haemoglobinopathies and genotypic markers of drug resistance. The study likewise wanted to investigate the applicability of drug efficacy trails in testing the importance of antibodies to different vaccine-candidates in patients receiving drugs with reduced efficacy, as suggested previously [14,22]. Patients were children below five years of age exposed to low-to-moderate levels of malaria transmission in Tanzania, treated with either SP or AQ for episodes of uncomplicated febrile malaria.

## Methods

### Study population and samples

The study was done as part of an annual clinical drug-efficacy trial under the East Africa Network for Monitoring Antimalarial Treatment (EANMAT) in collaboration with the National Malaria Control Programme in Tanzania. The trial was conducted during the rainy season between February and July 2005 in Chamwino village, Dodoma region, which is an area characterized by low-to-moderate malaria transmission of mainly *P. falciparum*. The study protocol was approved by the Ethical Committee of the National Institute for Medical Research and Ministry of Health, Tanzania.

The efficacy study were designed to enrol 100 patients aged 6–59 months presenting with uncomplicated malaria if they met the criteria as defined in the standard efficacy testing protocol by WHO [24]: *i) *monoinfection with *P. falciparum *at parasite densities above 2,000/μl and below 200,000/μl, *ii) *axillary temperature ≥ 37.5°C, *iii) *haemoglobin > 5 g/dl, *iv) *absence of severe malnutrition, *v) *absence of general danger signs, severe and complicated malaria, and *vi) *informed written consent from parents/guardians.

Patients were randomly allocated to receive either sulphadoxine-pyrimethamine (SP) (25 mg/kg sulphadoxine and 1.25 mg/kg pyrimethamine) as a single dose (Fansidar^®^, Roche, Switzerland) or amodiaquine (AQ) (10 mg/kg), once daily for three days (Pfizer, Senegal). Patients were followed up clinically and parasitologically on days 0, 1, 2, 3, 7, 14, and 28 and on any other day if the patient felt unwell.

Treatment outcome during 28 days of follow-up were classified as early treatment failure (ETF), late clinical failure (LCF), late parasitological failure (LPF) or adequate clinical and parasitological response (ACPR) according to the standard protocol of WHO [24]. For the purpose of this study, patients with ETF, LCF and LPF were grouped as treatment failures (TF). LPF were defined as patients with presence of parasitaemia on any day from day 7 to day 28 and axillary temperature < 37.5°C without previously having ETF or LCF. Patients failing treatment or with signs of severe malaria were given quinine and referred to the nearby district hospital.

Parasitaemia was determined microscopically using thin smear of fingerprick blood and stained with 3% Giemsa. Packed cell volume (PCV) was measured in haematocrit capillary tubes. Plasma samples were collected upon centrifugation, and stored at minus 20°C until further analysis.

### DNA and genetic analysis

DNA was extracted from fingerprick blood collected on filter paper, as previously described [25]. Nested polymerase chain reaction (PCR) assays were applied to differentiate recrudescence from new *P. falciparum *infections by comparing PCR-generated *msp2 *genotype patterns in regions FC27 and IC-1 from samples collected on day 0, 7, 14 and 28 [26]. Analysis of the *msp2 *genotypes was done according to Cattamanchi *et al *[27]: An outcome was defined as recrudescence if a subsequent sample on any day from day 7 to day 28 post-treatment contained identical alleles or a subset of the alleles present in the day 0 sample and the patient were classified as having late parasitological treatment failure (LPF). An outcome was defined as re-infection if a subsequent sample on any day from day 7 to day 28 post-treatment contained only new alleles compared to the day 0 sample and the patient were classified as having an adequate clinical and parasitological response (ACPR) [24].

*Plasmodium falciparum *single nucleotide polymorphisms (SNPs) at *dhfr *(position 50/51, 59 and 108), *dhps *(position 436/437, 540, 581 and 613) and *Pfcrt *(position 72–76) were determined on day 0 by sequence-specific oligonucleotide probes (SSOP) and Enzyme-Linked ImmunoSorbent Assay (ELISA)-based technique of PCR amplified fragments as described in [27] Briefly, biotin-conjugated nested PCR amplified DNA were fixed on streptavidin-coated ELISA plates and mixed with digoxigenin-labelled oligonucleotide probes with specificity for the SNP's of interest. The mixtures were washed with high stringency at set temperatures before incubated with peroxidase-conjugated anti-digoxigenin antibodies and visualized by o-phenylene-diamine (OPD). The SSOP's enables detection of mixed haplotypes with high specificity. The SNP's were constructed into haplotypes. Infections with mixed parasite haplotypes were only classified as 'mixed' when none of the polymorphisms in the same codon were dominant as determined by OD values of differences above 50%. Sickle cell trait were detected by screening the human β-haemoglobin gene for the A18T mutation by SSOP-ELISA [28] and the African alpha^3.7 ^deletion variant of alpha^+^-thalassaemia was determined as previously described [29].

### Recombinant *P. falciparum *antigens and synthetic peptides

The *P. falciparum *antigens used in the present study were: the N-terminal non-repeat R0 region of the Glutamate-rich Protein (GLURP-R0) (amino acids 27–500, FVO strain) [30]; the C-terminal GLURP-R2 repeat region (amino acids 705–1178, F32 strain) [31]; the synthetic peptide corresponding to the repeat region (NANPx6) of the circumsporozoite protein (CSP); the EBA-4 peptide of the Erythrocyte Binding Antigen-175 (EBA-175) [32]; the N-terminal region of the Apical Membrane Protein 1 (AMA1) (amino acids 25 to 545, FVO strain) with mutated glycosylation sites [33]; the non-polymorphic C-terminal region of the Merozoite Surface Protein 3 (MSP3) (amino acids 212–380, FVO strain) [30]; the 19 kDa fragment of Merozoite Surface Protein 1 (MSP1) [34]; three PfEMP1 recombinant HIS-tagged proteins: The CIDR1-α and DBL4-γ-DBL5-δ domains of the PFD1235w and the DBL2-β domain of the PF13_0003 [35,36]; and crude extract of schizont material [37].

### Antibody levels estimated by ELISA

The plasma samples collected at day 0 were tested for the presence of IgG to the eleven antigens mentioned above by ELISA as previously described [35]. Wells of Maxisorp microtiter plates (Nunc, Roskilde, Denmark) were coated with 100 μl of the recombinant protein diluted in 0.1 M glycine-HCl (pH 2.75) (apart from CSP that was diluted in phosphate-buffered saline [PBS], pH 7.2) by overnight incubation at 4°C. Plasma collected from 30 residents of a Tanzanian village where malaria is holoendemic were used as a reference positive plasma pool and plasma from 19 healthy Danish donors who have never been exposed to malaria were used as negative controls. To account for day-to-day variation between assays, antibody responses were calculated as arbitrary units (AU) with the following formula: [(OD_sample plasma _- OD_background_)/(OD_positive plasma _- OD_background_)] × 100. The cut-of for a positive antibody response was defined as the mean level plus two standard deviations of the antibody reactivity among the negative controls.

### Measurement of VSA-specific IgG by flow cytometry

Plasma levels of IgG with specificity for variant surface antigens (VSA) on two parasite isolate lines with different VSA-expression profiles, 3D7 unselected (VSA1) and 3D7 selected on transformed human bone marrow endothelial cells (THrMEB) (VSA2) [35] were tested by a flow cytometric assay previously described [38]. Plasma from six residents of a holoendemic Ghanaian village and six Danish donors served as positive and negative controls, respectively. For each plasma sample, the mean fluorescence index (MFI) was recorded and used as a measure of the VSA-specific antibody level. Antibody responses were calculated as arbitrary units (AU) with the following formula: [(MFI_sample plasma _- MFI_background_)/(MFI_positive plasma _- MFI_background_)] × 100. The antibody positivity cut-of was determined as the mean antibody levels of the plasma from the mean of the six Danish donors plus two standard deviations.

### Statistics

Statistical analyses of data were performed with Stata/SE version 8.2 (Stata Corp., Texas, US). χ^2 ^test and Fisher's exact test were used to compare differences in proportions of antibody responders, presence of parasite resistant genotypes and genes assessing haemoglobinopathies in patients with adequate clinical and parasitological response (ACPR) and treatment failure (TF). Non-parametric Mann Whitney rank sum test was used to analyse differences in median antibody levels between treatment groups. Associations between quantitative variables as age, parasite densities and haemoglobin levels were assessed by linear regression. For multivariable analysis, logistic regression models were used to assess predictors of treatment failure. In these models, parasite density (log parasite density pre-treatment), age and age-squared were used as continuous exposure variables, while presence or absence of parasite resistant genotypes, human antibodies and genes causing haemoglobinopathies were included as categorical exposure variables. Possible effect modification between the exposure variables was considered by testing for interaction between presence of parasite mutant haplotypes and IgG antibodies. P < 0.05 was defined as significant.

## Results

### Characteristics of the study groups and outcome of treatment

The baseline characteristics of the 100 patients enrolled in the study are shown in Table 1. Age, sex, parasitaemia, temperature and haemoglobin levels on the day of enrolment were similar in the SP and AQ treatment groups.

**Table 1:** Baseline characteristics of patients.

	**SP (n = 50)**		**AQ (n = 50)**	
		
**Treatment outcome**	**TF**	**ACPR**	**TF**	**ACPR**
PCR un-corrected, N (%)	35 (70)	15 (30)	22 (44)	28 (56)
PCR corrected, N (%)	34 (68)	16 (32)	19 (38)	31 (62)
Median age (months, CI)	31.5 (25.3–36)	27.5 (19–41.4)	24 (19.4–43.9)	32 (23.5–38.5)
Females (%)	31.6	38.7	41.2	68.7
Geometric mean parasite density, day0 (/ul. CI)	39.810 (28.184–56.234)*	17.865 (8.511–37.153)	28.840 (14.256–57.544)	32.359 (22.387–43.651)
				
Mean haemoglobin day0 (PCV, SD)	29.4 (4.8)	29.6 (5.8)	28.6 (6.5)	30.7 (5.1)
Mean temperature day0 (°C. SD)	38.8 (0.9)	38.5 (0.6)	38.5 (0.9)	39.1 (0.8)
Alpha^+^-thalassaemia, %	33.4	27.6	42.9	35.7
HbAS, %	15.2	12.5	10.4	9.7

TF: Parasitological or clinical treatment failure, ACPR: Adequate clinical and parasitological response. SP: sulphadoxine-pyrimethamine, AQ: amodiaquine. * Significantly different from value for parasite density in ACPR group as calculated by Student's t test.

Of the 50 patients receiving SP, none presented with early clinical failure (ECF), three patients presented with late clinical failures (LCF), 31 patients presented with late parasitological failures (LPF) and the remaining 16 patients had an adequate clinical and parasitological response (ACPR). Of the 50 patients receiving AQ, one presented with ECF, two patients presented with LCF, 16 patients presented with LPF and the remaining 31 patients had an ACPR. All samples were adjusted for new infections by PCR genotyping. Before PCR corrections, 32 patients receiving SP and 22 patients receiving AQ presented with LPF, respectively. All patients with either ECR, LCF or LPF were grouped together as treatment failures (TF). Thus, the treatment failure rates were 68% in the SP group and 38% in the AQ group. There were no significant differences with respect to age, sex, temperature, and haemoglobin level between patients with ACPR or TF in any of the treatment groups. However, in patients receiving SP, the parasite density on day 0 was higher in the TF than ACPR group (P = 0.03). Levels of haemoglobin, parasite density and failure rates showed no relation to age (data not shown).

### Associations between parasite mutant haplotypes and treatment outcome

The haplotypes based on the single nucleotide polymorphisms at codon 50/51, 59 and 108 of the *P. falciparum dhfr *gene, and codon 436/437, 540, 581 and 613 of the *P. falciparum dhps *gene, were determined on day 0 for parasite infections in patients receiving SP (Table 2).

**Table 2:** Frequency of *P. falciparum *polymorphisms in *dhfr, dhps *and *crt *genes in relation to treatment outcome

**SP**	**Haplotypes**	**TF**	**ACPR**
N		34	16

*dhfr*			
wildtype	CNCS	0	2
S108N, single	CNCN	1	1
C59R + S108N, double	CNRN	1	1
N51I + S108N, double	CICN	3	3
N51I + C59R + S108N, triple	CIRN	28	8
Mixed*		1	1

*dhps*			
wildtype	SAKAA/AAKAA	18	11
A437G, single	SGKAA	5	0
A437G + K540E, double	SGEAA	11	4
single-double combined		16	4
Mixed*		0	1

*dhfr-dhps*			
triple-double		10	3
triple-single		4	0
double-double		1	1

**AQ**		**TF**	**ACPR**

N		19	31

76K	CVNMK	3	12
76T	CVIET	16	18
Mixed*		0	1

TF: Parasitological or clinical treatment failure, ACPR: Adequate clinical and parasitological response. SP: sulphadoxine-pyrimethamine, AQ: amodiaquine. Haplotypes defined as amino acid sequences based on the following substitutions in *dhfr*: S108N (single), C59R + S108N or N51I + C59R (double) and N51I + C59R + S108N (triple), and in *dhps*: A437G (single) and A437G + K540E (double) were assessed. For the *crt *gene, the K76T amino acid substitution was assessed. *Classified as mixed when none of the haplotypes were dominant.

A higher prevalence of the triple mutant haplotype, CIRN in *dhfr*, was observed in patients experiencing TF (82%) compared to patients with ACPR (50%), (χ^2 ^= 5.6, P = 0.02). There was no significant association between *dhps *mutant haplotypes (single SGKAA and double SGEAA combined) and treatment outcome (χ^2 ^= 1.8, P = 0.18), nor between the quintuple *dhfr*-*dhps *mutant haplotype (CIRN-SGEAA) and TF (χ^2 ^= 0.6, P = 0.46). However, due to the low number of samples, we could not exclude a possible association between the quintuple mutant haplotype and treatment outcome.

The majority of parasites in patients receiving AQ expressed the *Pfcrt *mutant haplotype CVIET (68%) and the remaining samples were the CVMNK wildtype haplotype. There was a trend towards higher prevalence of the CVIET haplotype in patients failing treatment (84%) compared to patients with ACPR (58%), although not statistically significant (χ^2 ^= 3.2, P = 0.08).

Thus, a higher prevalence of parasites expressing triple mutant *dhfr *haplotype in patients failing SP treatment and a tendency for a higher prevalence of the mutant CVIET haplotype in patients failing AQ treatment were observed, indicating some association between presence of parasite resistant genotypes and treatment outcome.

### Associations between anti-malaria antibodies and treatment outcome

**Figure 1:**
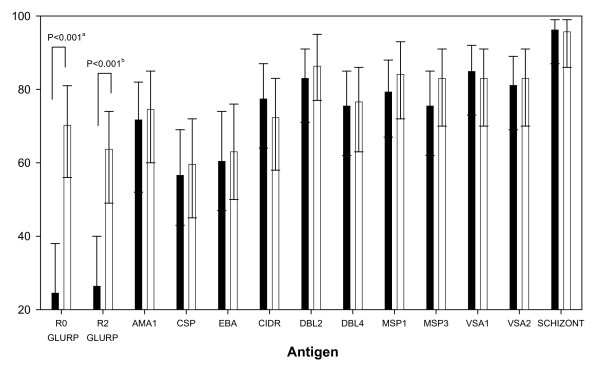
**Prevalence of IgG antibody in patients at day 0 to different malaria antigens in relation to treatment outcome**. Black: Parasitological or clinical treatment failure (TF), White: Adequate clinical and parasitological response (ACPR). Patients receiving sulphadoxine-pyrimethamine (SP) or amodiaquine (AQ) are grouped together. Error bars illustrate 95% confidence intervals. χ^2 ^test were done to compare proportions of IgG antibody responders between treatment outcome groups. Only significant p-values are presented. ^a^GLURP-R0; χ^2 ^= 20.9, ^b^GLURP-R2; χ^2 ^= 12.7. GLURP-R0: Glutamate-rich Protein region N-terminal, GLURP-R2: Glutamate-rich Protein region C-terminal, AMA1: Apical Membrane Antigen 1, CSP: Circumsporozoite Protein, EBA: Erythrocyte Binding Antigen-175, CIDR: CIDR domain of PfEMP1 (PFD1235w), DBL2: DBL2 domain of PfEMP1 (PF13_0003), DBL4: DBL4 domain of PfEMP1 (PFD1235w), MSP1: Merozoite Surface Protein 1, MSP3: Merozoite Surface Protein 3, VSA1: Variant Surface Antigen on unselected 3D7 parasite strain, VSA2: Variant Surface Antigen on bone marrow selected 3D7 parasite strain. SCHIZONT: Crude extract of schizont material.

Prevalence and levels of IgG antibodies with specificity for the eleven malaria antigens and the surface of the two parasite isolates were measured on day 0 in patients treated with SP or AQ (Figure 1 and Table 3). In univariate analysis, patients with ACPR had a higher prevalence of anti-GLURP-R0 IgG (χ^2 ^= 20.9, P < 0.001) and anti-GLURP-R2 IgG (χ^2 ^= 12.7, P < 0.001) as well as higher levels of anti-GLURP-R0 and R2 IgG (P < 0.001) compared to patients with TF. Such statistically significant associations were not observed for any of the other antigen-specific IgG responses. Furthermore, the associations between ACPR and prevalence and levels of GLURP-R0 and R2 antibodies were significant when the SP and AQ groups were analysed separately ([SP]: R0: χ^2^= 11.8, P = 0.001; R2: χ^2 ^= 9.4, P = 0.002. [AQ]: R0: χ^2 ^= 11.3, P = 0.001; R2: χ^2 ^= 3.3, P = 0.07). The prevalence of the combined IgG responses to GLURP-R0 and R2 was also correlated to ACPR in both the SP (χ^2 ^= 10.1, P = 0.002) and the AQ (χ^2 ^= 11.3, P = 0.001) group. By contrast, no association between antibody responses to any other antigen, analysed individually or in combination, and treatment outcome in neither the SP nor the AQ group was demonstrated. Approximately 25% of patients with IgG against GLURP R0 did not have IgG against R2 and vice versa, indicating that responding to R0 not necessarily means responding to R2. Thus, among the thirteen IgG specificities, only anti-GLURP-R0 and R2 IgG proved to be associated with ACPR.

**Table 3:** Level of IgG antibodies in patients to different malaria antigens in relation to treatment outcome.

	Median level (25^th ^and 75^th ^percentiles)*		
		
Antigen	TF, n = 53	ACPR, n = 47	P-value
GLURP-R0	0.0 (0.0–1.3)	8.4 (0.0–47.7)	< 0.001
GLURP-R2	0.0 (0.0–5.0)	7.7 (0.0–49.8)	< 0.001
AMA-1	24.3 (0.0–51.1)	24.5 (0.0–87.3)	0.25
CSP	5.8 (0.0–17.1)	10.2 (0.0–24.4)	0.42
CIDR1α	15.4 (3.3–61.7)	9.1 (0.0–30.2)	0.24
DBL2β	22.5 (6.9–45.6)	16.9 (3.4–58.3)	0.92
DBL4γ-DBL5δ	10.4 (1.6–38.6)	12.4 (3.0–44.0)	0.74
MSP-1	80.0 (6.7–144.4)	122.7 (29.3–162.0)	0.11
MSP-3	5.3 (1.1–12.6)	8.0 (2.5–18.9)	0.30
VSA1	15.5 (5.2–33.0)	19.8 (6.1–41.0)	0.58
VSA2	16.3 (4.1–30.9)	21.1 (7.6–38.7)	0.21
EBA-175	4.3 (0.0–14.1)	5.9 (0.0–21.0)	0.54
SCHIZONT	30.0 (12.9–51.0)	35.2 (11.1–80.2)	0.17

TF: Parasitological or clinical treatment failure, ACPR: Adequate clinical and parasitological response. Patients receiving sulphadoxine-pyrimethamine (SP) or amodiaquine (AQ) are grouped together. *Median values of IgG level day 0 in arbitrary units (25^th ^and 75^th ^percentiles). P-values determined by Mann-Whitney rank-sum test. Similar tendencies were observed when restricting the analysis to those with a measurable antibody level only, and when analysing each drug arm separately. For definitions of antigens, see Figure 1 and in the method section.

### Impact of haemoglobinopathies on anti-malaria antibodies, parasitaemia and treatment outcome

In the study population, the overall frequency of homozygote and heterozygote alpha^+^-thalassaemia was 38% and the frequency of sickle cell trait (HbAS) 12%. No sickle cell anaemic patients (HbSS) were found. The prevalence of these traits was similar in the two treatment outcome groups for both SP and AQ (see Table 1). Prevalence and levels of antibodies did not differ between patients with and without sickle cell trait or with and without alpha^+^-thalassaemia for any of the malaria antigens. A lower parasite density was observed at enrolment in patients with alpha^+^-thalassaemia receiving AQ (P = 0.007), but not for SP (P = 0.41).

### Potential predictors of treatment outcome

Patients were divided into four groups based on infections with or without mutant haplotypes (the *dhfr *triple mutant haplotype in the SP group, and the CVIET haplotype in the AQ group), and presence or absence of IgG antibodies to each antigen. In Table 4, only data for GLURP-R0 and R2 are shown.

**Table 4:** Associations between presence of IgG antibodies to GLURP-R0 + R2 and parasite haplotypes in relation to treatment outcome.

		GLURP-R0		GLURP-R2	
			
Treatment and group SP	Presence of antibody/resistant haplotype*	TF	ACPR	TF	ACPR
1	+/-	2	4	1	6
2	-/-	4	3	5	2
3	+/+	6	7	9	7
4	-/+	22	2	19	1

AQ					

1	+/-	1	7	0	8
2	-/-	2	5	3	4
3	+/+	4	12	3	11
4	-/+	12	6	13	7

TF: Parasitological or clinical treatment failure, ACPR: Adequate clinical and parasitological response. SP: sulphadoxine-pyrimethamine, AQ: amodiaquine. *Number of patients with presence (+) or absence (-) of IgG antibodies to GLURP-R0 or R2 combined with resistant (+) or sensitive (-) genotypes associated with treatment outcome. Haplotypes were defined as resistant if parasites expressed the *dhfr *triple haplotype (CIRN) for the SP group or the CVIET haplotype for the AQ group.

As expected, patients with anti-GLURP IgG infected with a sensitive haplotype (group 1) were more likely to have ACPR compared to patients with no anti-GLURP IgG infected with a resistant parasite haplotype (group 4). Interestingly, there was a marked positive effect of having anti-GLURP IgG on treatment outcome in patients with resistant haplotypes (comparing group 3 vs. 4 for GLURP-R0; [SP]: R0, P = 0.004 and [AQ]: R0, P = 0.02), indicating that possessions of anti-GLURP IgG were associated with reduced risk of TF when infected with resistant parasites. Similar significant associations were seen for GLURP-R2 but not for any of the other antigens.

In multivariate regression models, the effect of IgG antibodies, parasite haplotype and parasite density on treatment outcome was analysed (Table 5). Regardless of parasite density and age, anti-GLURP IgG were the strongest predictor of treatment outcome in the SP and AQ groups separately and combined. Presence of resistant parasite haplotypes and high parasitaemia were also associated with treatment failure in patients receiving SP, but not as strongly as the presence of GLURP-R0 and R2 IgG and not in the AQ group.

**Table 5:** Predictors for treatment failure.

Treatment group	Risk variables	Unadjusted OR (95% CI)	p-value	Adjusted OR (95% CI)*	p-value
SP (n = 50)	Parasite density	3.74 (1.12–12.54)	0.03	3.16 (0.73–13.71)	0.13
	CIRN	4.67 (1.25–17.44)	0.02	7.21 (1.42–36.73)	0.02
	GLURP-R0	0.09 (0.02–0.41)	0.002	0.08 (0.14–0.47)	0.005

AQ (n = 50)	Parasite density	0.82 (0.27–2.46)	0.72	0.77 (0.13–4.42)	0.76
	CVIET	3.55 (0.85–14.91)	0.08	2.56 (0.46–14.27)	0.28
	GLURP-R0	0.10 (0.02–0.43)	0.002	0.07 (0.01–0.48)	0.003

SP+AQ (n = 100)	Parasite density	1.61 (0.74–3.47)	0.23	1.13 (0.43–2.99)	0.80
	CIRN/CVIET	3.76 (1.49–9.47)	0.005	3.44 (1.12–10.54)	0.03
	GLURP-R0	0.13 (0.06–0.33)	< 0.001	0.11 (0.04–0.31)	< 0.001

Multivariate regression models predicting failure of antimalarial treatment. SP: sulphadoxine-pyrimethamine, AQ: amodiaquine.OR: Odds Ratio, CI: 95% Confidence interval. CIRN: Presence of the triple *dhfr *CIRN haplotype. CVIET: Presence of the *crt *CVIET haplotype. CIRN/CVIET: Presence of parasites with CIRN in patients treated with SP and presence of parasites with CVIET in patients treated with AQ. GLURP-R0: Presence of IgG antibodies to GLURP-R0. SP+AQ: Total patients in the two treatment groups. *Adjusted for age, parasite density, presence of mutant haplotypes and GLURP-R0 antibodies. No interaction between presence of mutant haplotypes and GLURP-R0 IgG antibodies was detected.

## Discussion

This study evaluated the contribution of some anti-malaria antibodies and parasite resistant haplotypes on the outcome of SP and AQ treatment of patients under five years of age with uncomplicated malaria. The rate of SP treatment failure observed (68%) is unexpectedly high for this area of endemicity, but similar SP failure rates (43%–74%) have been shown in other parts of Tanzania the last 10 years [39-41]. Not surprisingly patients failing SP and AQ treatment were generally infected with parasites expressing drug resistance-related mutations. However, increased risk of SP treatment failure was associated with the triple *dhfr *(CIRN) irrespective of the *dhps *haplotype status as demonstrated in other studies [5,42]. In the AQ treatment group, only a trend towards an increased risk of treatment failure was found in patients infected with the *Pfcrt*-CVIET mutant haplotype. Although the latter could be explained by involvement of other putative genes related to AQ resistance, such as *Pfmdr *[43], and that the assumptions are based on a relatively small sample size, these findings indicate that the association between parasite resistant genotypes and SP or AQ treatment outcome is not absolute.

Interestingly, the prevalence and level of anti-GLURP antibodies was associated with a substantial decreased risk of treatment failure irrespective of drug regimen. This association could not be demonstrated for any of the other antigens studied, neither individually nor combined. Similarly, although there was an association between parasite density and patients failing SP treatment, the treatment outcome was independent of age, alpha^+^-thalassaemia (homozygote or heterozygote) and sickle cell trait, and for AQ treatment outcome, also parasite density. Furthermore, in regression analysis, the presence of GLURP R0 or R2 specific IgG antibodies influenced the risk for failing SP and AQ treatment more strongly than presence of parasite resistant haplotypes. This is in line with a recent study from Uganda, where transmission intensity, rather than the parasite genotype, influenced treatment outcome [16]. This indicates that the degree of immunity in the population in combination with the number of resistant-related parasite mutations predicts ACPR, suggesting a synergistic effect between the antimalarial and host immunity, as also demonstrated in mice [17].

It has recently been proposed that therapeutic responses to resistant malaria infections can provide a mechanism for measuring effective clinical immunity [14], i.e. drug trials can be useful in identifying antigens and vaccine candidates affording protection [22]. Investigations of the impact of immunity on treatment outcome have however resulted in conflicting findings [19-23]. In line with the observations from the present study, level of anti-IgG to MSP1_19 _was not associated with CQ [22] SP [23] or AQ [21,23] treatment outcome, and prevalence of anti-AMA1 [22] and anti-MSP3 [19] IgG was not associated with reduced risk for CQ treatment failure. However, in contrast to the findings from the present study, the prevalence of IgG to MSP1_19 _was higher in patients clinically recovering after CQ treatment [22], and IgG reactivity to multiple, but not single, K1 and MAD20 alleles of MSP1 were related to efficacy of AQ treatment [21]. In addition, prevalence of anti-NANP and anti-RESA IgG has been demonstrated to be higher in patients recovering successfully from CQ [19] and artesunate [20] treatment. The inability to demonstrate an association between prevalence and levels of antibodies against antigens other than GLURP in the present study may be explained by the relatively small sample size (50 patients in each group); alternatively by allelic diversity as shown in the study from Gabon (20), or by variable IgG subclass composition of the patient sera. For instance, it has previously been described that only IgG3 against MSP3 is associated with protection against clinical malaria [44], although this was not observed in another study where total IgG was a strong predictor of protection [45]. Finally, one could speculate if prevalence or level of IgG antibodies to these other specific antigens are not primary responsible for, or associated with, host immune protection against uncomplicated malaria but rather severe malaria – which none of the patients had. However, in patients with established clinical malaria, sporozoite-blocking anti-CSP antibodies were, however, not expected to influence treatment outcome.

Interestingly, at day 0, all patients had IgG antibodies to at least one of the antigens tested, but no patient had IgG antibodies to all the antigens. This may reflect substantial variation in response to infections. Previous immuno-epidemiological studies have demonstrated that GLURP-specific IgG antibodies are associated with protection against high parasitaemia [46], clinical disease [47] and inhibition of parasite growth in vitro [48], but this is not direct evidence of IgG antibodies against GLURP increase the ability to clear uncomplicated malaria infections, nor of a causal relationship between treatment success and IgG antibodies against GLURP. However, it was shown that patients clinically recovering after treatment are more likely to carry anti-GLURP IgG antibodies and the presence of such antibodies were associated with reduced risk of failing drug-resistant parasite infections.

## Conclusion

This study suggests that presence or level of GLURP-specific IgG antibodies in some settings are better predictors of SP and AQ treatment outcome than the parasite density at enrolment and drug-resistant related parasite mutations. This confirms the hypothesis that acquired immunity enhances the efficacy of antimalarial treatment, and supports the use of drug trials to identify markers of immunological importance. In addition, the findings suggest that drugs with reduced efficacy, such as SP, may still be effective for treatment of uncomplicated malaria in individuals with some acquired immunity, such as older children and adults living in areas of intense malaria transmission.

## Authors' contributions

AE, LV, MT and MA conceived and designed the study. WN was responsible for the clinical trial and the supervision of patient enrolment and sample collection. AE performed the experiments and analysis with MA, MT, IB and LV. TS, AJ, IB and TT participated in manuscript preparation and design of the study. All authors read and approved the final manuscript.
